# Prevalence, risk factors and trends of human schistosomiasis in Côte d’Ivoire from 1974–2023: systematic review and meta-analysis

**DOI:** 10.1186/s40249-025-01410-9

**Published:** 2026-01-12

**Authors:** Rufin K. Assaré, Fidèle K. Bassa, Jean T. Coulibaly, Nana R. Diakité, Mamadou Ouattara, Eliézer K. N’Goran, Jürg Utzinger

**Affiliations:** 1 Unité de Formation et de Recherche Biosciences, Université Félix Houphouët-Boigny, 22 BP 582, Abidjan 22, Abidjan, Côte d’Ivoire; 2 Centre Suisse de Recherches Scientifiques en Côte d’Ivoire, Abidjan 01, 01 BP 1303 Abidjan, Côte d’Ivoire; 3 Swiss Tropical and Public Health Institute, Kreuzstrasse 2, CH-4123 Allschwil, Switzerland; 4 University of Basel, CH-4003 Basel, Switzerland

**Keywords:** Côte d’Ivoire, Meta-analysis, Prevalence, Risk factor, Schistosomiasis, Systematic review

## Abstract

**Background:**

Schistosomiasis is a parasitic worm infection that affects an estimated 250 million people. In Côte d’Ivoire, schistosomiasis remains a public health problem despite control efforts that have been mounted since the new millennium. The aim of this study was to assess the pooled prevalence of human schistosomiasis, to determine trends over the past 50 years and to identify risk factors for schistosomiasis.

**Methods:**

We systematically searched Google Scholar, PubMed, Scopus and Web of Science Core Collection without language restriction for papers published from January 1, 1974 to December 31, 2023. We adhered to Preferred Reporting Items for Systematic Reviews and Meta-analysis guidelines. We performed random effect models for meta-analysis and generated forest plots. Pooled schistosomiasis prevalences and corresponding 95% confidence intervals (*CI*s) were determined. Heterogeneity among studies were evaluated using Cochran’s *Q* test and *I*^2^ statistic test. Publication bias was assessed with funnel plot and Egger’s test.

**Results:**

Overall, 326 articles involving 279,340 participants were included, comprising 254,954 school-aged children and 520 preschool-aged children. The pooled prevalence of schistosomiasis was 26.1%. The prevalence decreased from 66.5% in 1994–2003 to 15.0% in 2014–2023. The highest pooled prevalence of schistosomiasis was observed in Tonkpi regional health directorate. The main risk factors for schistosomiasis were sex [male: odds ratio (*OR*) = 1.24, 95% *CI:* 1.13–1.35], age group (> 15 years:* OR* = 2.45, 95% *CI:* 1.82–3.08, compared to children aged 6–10 years), and altitude (< 400 m,* OR* = 4.76, 95% *CI:* 4.00–5.88).

**Conclusion:**

Our findings revealed that the prevalence of schistosomiasis in Côte d’Ivoire has considerably declined over the past decades. However, the disease remains a public health problem, and hence, surveillance should be tightened up and control efforts targeted to high-risk communities.

**Graphical Abstract:**

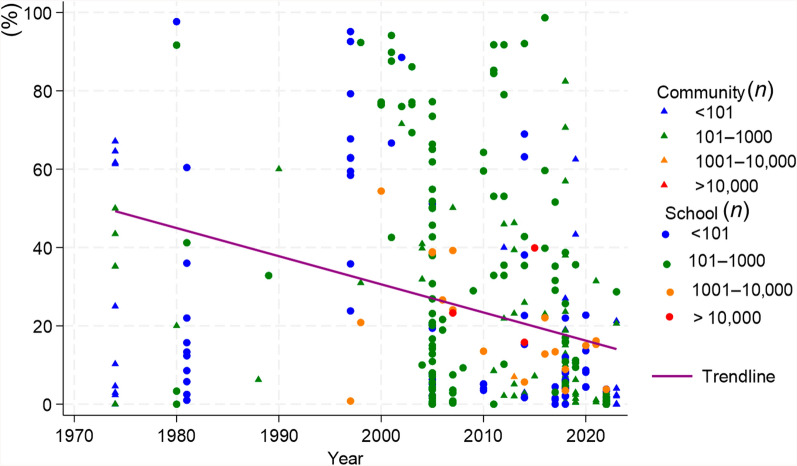

**Supplementary Information:**

The online version contains supplementary material available at 10.1186/s40249-025-01410-9.

## Background

Schistosomiasis is endemic in 78 countries and constitutes a public health problem mainly in tropical and sub-tropical areas [1]. Schistosomiasis can lead to bladder cancer, infertility, hepatomegaly and splenomegaly [2–5]. In children, schistosomiasis is associated with anaemia, poor physical and delayed cognitive development. In 2013, an estimated 250 million people were infected worldwide with more than 80% concentrated in sub-Saharan Africa [6]. In 2021, the prevalence of schistosomiasis across Africa was estimated at 9.4% [7]. In Africa, schistosomiasis is mainly due to *Schistosoma haematobium* and *S. mansoni*. The latter causes intestinal schistosomiasis, while *S*. *haematobium* causes urogenital schistosomiasis. Hybridization within human schistosome species and between human and animal schistosomes have been reported in the western, central and southern parts of the continent, which highlight a potential zoonotic transmission of schistosomiasis [8–11]. Preventive chemotherapy with praziquantel is the mainstay of schistosomiasis control [12]. This strategy is pivotal for morbidity control and might also impact on transmission. However, re-infection can occur rapidly in the absence of ancillary measures. Hence, there is a need for integrated control approaches by combining preventive chemotherapy with provision of adequate water, sanitation and hygiene (WASH) facilities, behaviour change, communication and health education, and snail control [13–15].

In Côte d’Ivoire, schistosomiasis has been reported since the 1950s [16] and control efforts have been mounted by the Ivorian government to reduce the burden of the disease [17]. The first national schistosomiasis control programme was established in 1994 and included simultaneously human African trypanosomiasis and onchocerciasis within the frame of the West African Onchocerciasis Control Programme [17]. In 1998, the “Programme National de Lutte contre la Schistosomiase, les Géohelminthiases et la Filariose Lymphatique” was launched [17]. In 2012, the government of Côte d’Ivoire endorsed the London Declaration on Neglected Tropical Diseases and supported the World Health Organization (WHO) roadmap to prevent, control and eliminate schistosomiasis and other neglected tropical diseases. For effective implementation of preventive chemotherapy against parasitic worm infection in Côte d’Ivoire, the “Programme National de Lutte contre les Maladies Tropicales Negligées à Chimiothérapie Préventive” (PNLMTN-CP) was established in 2015, placing particular emphasis on schistosomiasis, soil-transmitted helminthiasis, onchocerciasis, lymphatic filariasis and trachoma.

Numerous parasitological assessments were carried out in Côte d’Ivoire since the 1970s to deepen the understanding of the epidemiology of schistosomiasis and to tailor control strategies to specific settings and population groups [14, 18, 19]. For instance, questionnaires, microscopic and antigen-based diagnostic approaches were evaluated for their accuracy in low-, moderate- and high-endemic settings [18, 20–22]. Recent studies showed that antigen-based methods might result in up to five-fold higher prevalence estimates of *S. mansoni* than the widely used Kato-Katz thick smear technique [23]. Several studies revealed that examination of repeated stool samples considerably increase the sensitivity of the Kato-Katz thick smear technique [24–26]. The Kato-Katz and urine filtration methods are the most widely used diagnostic approaches in research projects, such as mapping of schistosomiasis at different spatial scales, clinical trials to assess the efficacy of antischistosomal drugs and identification of disease risk factors [13, 27]. It was reported that socio-demographic, environmental and climatic factors, such as age, sex, socioeconomic status and altitude, might influence the occurrence, distribution and severity of schistosomiasis [28, 29]. Despite considerable efforts at administrative and research levels, the pooled prevalence and trends of schistosomiasis over time at the national level are poorly understood. In addition, prior studies often differed in terms of specific objectives, target population, sample sizes and diagnostic approach, among others.

The aim of this study was to estimate the pooled prevalence of schistosomiasis and the spatial distribution in the 20 regional health directorates of Côte d’Ivoire, and to assess trends over the past 50 years. Additionally, we determined risk factors of schistosomiasis. Results of this study will be useful for the PNLMTN-CP and schistosomiasis control and elimination efforts elsewhere in sub-Saharan Africa.

## Methods

### Protocol and registration

The protocol of this systematic review and meta-analysis was registered and is published on PROSPERO (https://www.crd.york.ac.uk/PROSPERO/view/CRD42021236785). The registration number is CRD42021236785. The following research question guided our review: What is the pooled prevalence of schistosomiasis in Côte d’Ivoire and how did it evolve over the past 50 years?

### Search strategy

We systematically searched four readily available electronic databases (i.e. Google Scholar, PubMed, Scopus and Web of Science Core Collection) for articles published from January 1, 1974 to December 31, 2023 without language restriction. In Google Scholar, the search terms were “Côte d’Ivoire”, “schistosomiases*”*, “schistosomiasis”, “prevalence” and “bilharziasis” for the period 1974–2023. The following search string ((schisto*) OR (bilharzi*)) AND (Côte d’Ivoire) (filters: from 1974–2023), TITLE-ABS-KEY-AUTH ((Côte AND dʼIvoire) AND (schistosomiase OR schistosomiasis OR prevalence OR bilharziasis)) AND (EXCLUDE (PUBYEAR, 2024)) and TS = ((Côte dʼIvoire) AND (schistosomiase, OR schistosomiasis OR prevalence OR bilharziasis)) refined by: [excluding] PUBLICATION YEARS: (2024) were utilized in PubMed, Scopus and Web of Science Core Collection. The systematic review and selection of relevant literature were performed according to the Preferred Reporting Items for Systematic Reviews and Meta-analysis (PRISMA) guidelines [30] (Additional file 1).

### Inclusion and exclusion criteria

Studies were included if they (i) pursued a cross-sectional design; (ii) were school-based and/or community-based; (iii) were conducted in Côte d’Ivoire; (iv) assessed human schistosomiasis prevalence and/or risk factors; (v) had available data that could be used to evaluate schistosomiasis prevalence; and (vi) employed the Kato-Katz thick smear technique for *S. mansoni* or urine filtration for *S. haematobium* diagnosis.

Studies were excluded if they (i) were carried out in health facilities; (ii) were review articles; (iii) consisted of comments, letters, conference abstracts or interviews; (iv) were based on secondary data; and (v) reported prevalence of *Schistosoma* infection in non-human vertebrate hosts. Authors of original studies were not contacted for additional information. In case a publication reported more than one unique study, each study was considered separately [31].

### Data extraction

One researcher performed the literature search (RKA). Articles were double-checked by another researcher (EKN), according to study inclusion and exclusion criteria. In case of discrepancies, a third researcher (JU) was involved and results discussed until consensus was reached. From each eligible study, the following information was extracted: title of the study, first author, year of publication, study location, health district, regional health directorate, study population (school-based or community-based), sample size, time of the study, diagnostic method, number of individuals who provided urine samples, number of *S. haematobium-*positive individuals, prevalence of *S. haematobium*, number of individuals who provided stool samples, number of *S. mansoni*-positive individuals and prevalence of *S. mansoni*.

### Quality assessment

The quality of studies included was assessed by using the checklist of Joanna Briggs Institute (JBI) for prevalence studies quality assessment tools. The assessment tool used the following nine items: (i) sample frame appropriate to address the target population; (ii) study participants sampled in an appropriate way; (iii) adequate sample size; (iv) study participants and setting described in detail; (v) data analysis conducted with sufficient coverage of the identified sample; (vi) valid methods used for the identification of the condition; (vii) the condition was measured in a standard and reliable way for all participants; (viii) appropriate statistical analysis; and (ix) adequate response rate. The response of each parameter was scored 0 for “not reported” and 1 for “reported”. Total scores ranged between 0 and 9 [32].

### Statistical analysis

Extracted data were entered into an Excel 2016 spreadsheet. Statistical analyses were performed using STATA version IC15.1 (Stata Corporation; College Station, TX, USA). Schistosomiasis prevalence estimates were reported with their corresponding 95% confidence interval (*CI*). For studies with number of participants examined and number of positive individuals, prevalence of schistosome species were estimated by multiplying the ratio of cases to sample size by a factor of 100. The 95% *CI* of schistosome prevalence was calculated using the exact binomial interval (http://statpages.info/confint.html accessed from 31 October 2023 to 31 January 2024).

Schistosomiasis risk factors were determined and expressed with odds ratio (*OR*). The relationship between schistosomiasis and the risk factors were estimated based on the pooled *OR*s. Statistical heterogeneity among the studies were assessed by Cochran’s *Q* test and *I*^2^ statistic test. *I*^2^ values of 25%, 50% and 75% were interpreted as low, moderate and high heterogeneity, respectively [33]. Cochran’s *Q* test with* P*-value (*P*) < 0.1 was considered to show statistically significant heterogeneity between studies [34]. Random effect models for the meta-analysis were used and forest plots generated. Forest plot, funnel plot and Egger’s test were generated using STATA version IC15.1 (Stata Corporation; College Station, TX, USA).

### Subgroup analysis, heterogeneity and publication bias

Subgroup analysis was done based on study setting (school or community), regional health directorates, schistosome species (*S. haematobium* and *S. mansoni*) and study decade (1974–1983, 1984–1993, 1994–2003, 2004–2013 and 2014–2023). Publication bias was statistically examined with funnel plot and Egger’s test [35]. A *P* < 0.05 of the Egger’s test was considered as indication for the presence of publication bias.

## Results

### Description of included studies

**Fig. 1:**
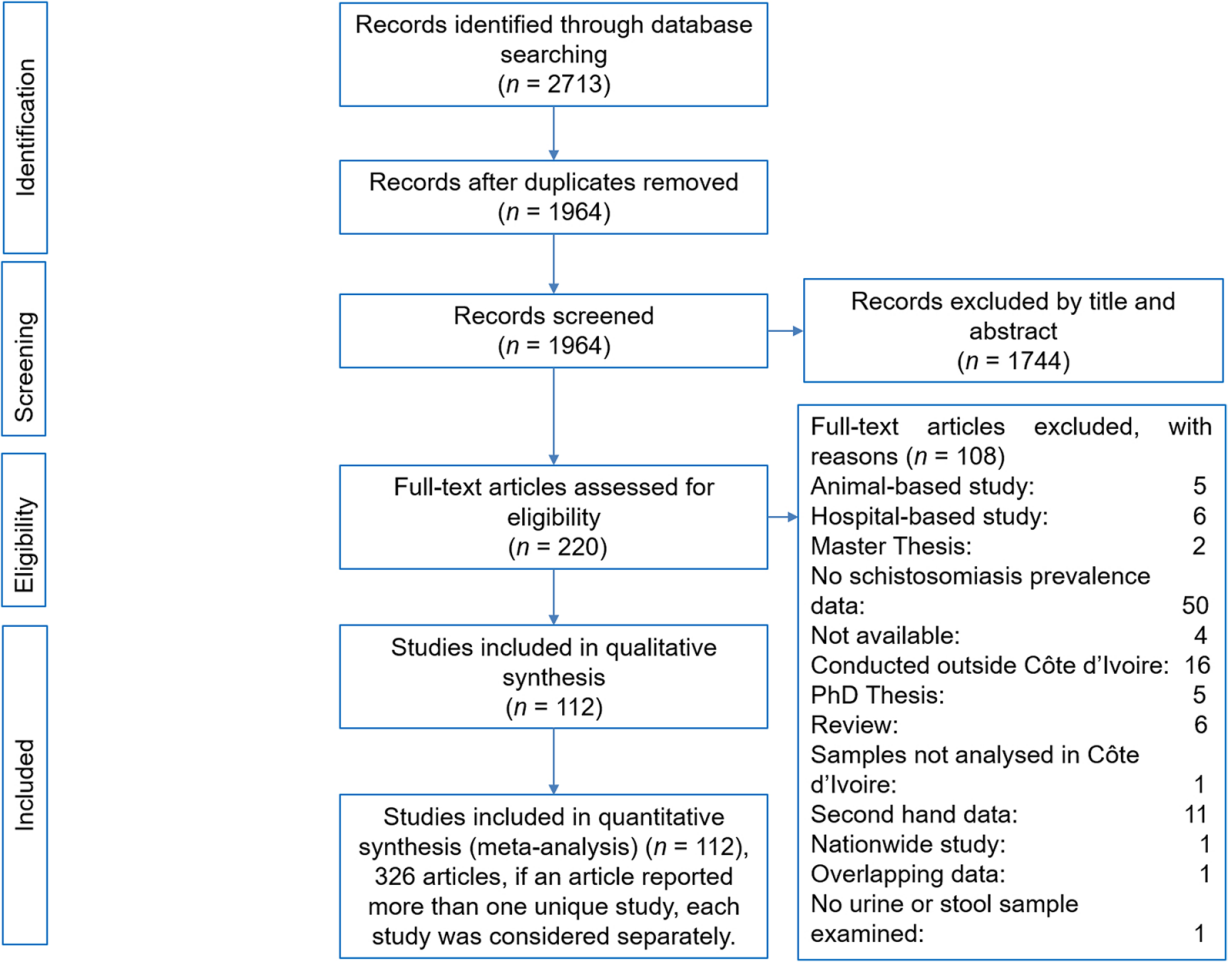
Flowchart of the study selection. *n*: number

Overall, 2713 articles were identified in the four electronic databases. After removing duplicates (*n* = 749) and non-relevant articles (*n* = 1852), 112 articles consisting of 326 unique studies fulfilled our inclusion criteria (Fig. 1). Among the 112 included articles, 96 articles (85.7%) and 13 (11.6%) were categorized as high quality and low quality, respectively (additional file 2).

### Study characteristics

All included studies had a cross-sectional design (additional file 3). Overall, 279,340 participants were included to estimate the pooled prevalence of schistosomiasis in Côte d’Ivoire. The sample size varied from 29 to 118,327 participants. The highest prevalence of schistosomiasis (98.7%) was reported in Azaguié Makouguié in Agboville health district belonging to Agnéby-Tiassa Mé regional health directorate. The lowest prevalence of infection (0.0%) was observed in 26 locations.

**Fig. 2:**
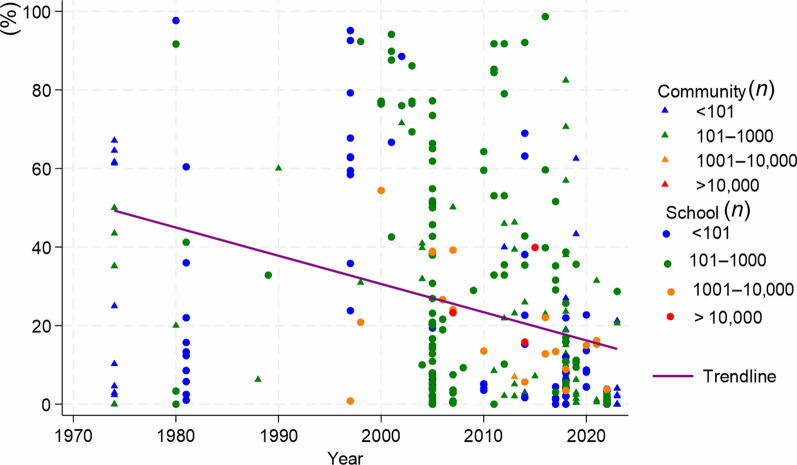
Reported schistosomiasis prevalences in community and school-based studies from 1974 to 2023 in Côte d’Ivoire. *n*: sample size

**Fig. 3:**
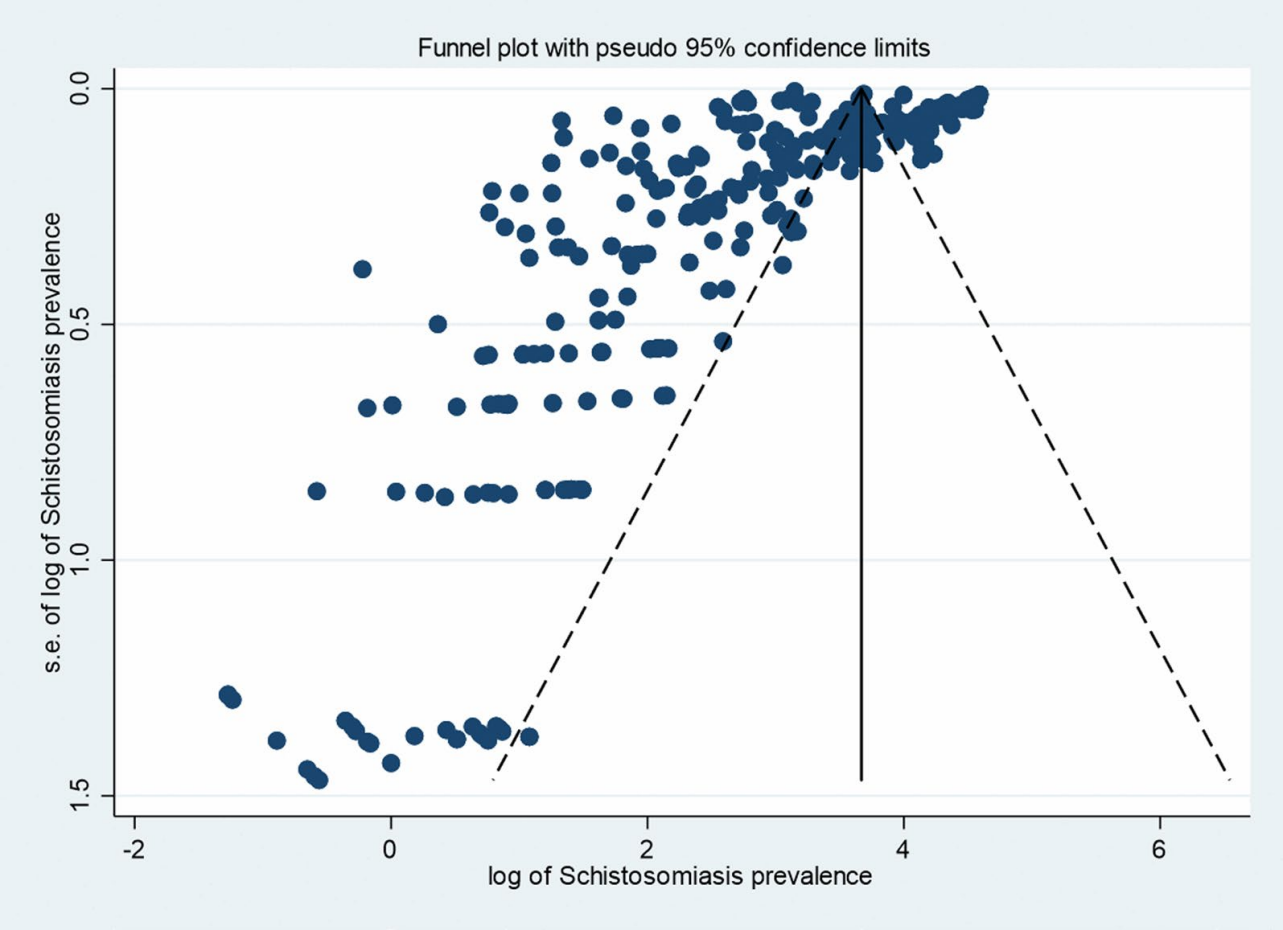
Funnel plot of schistosomiasis studies. Egger’s test [*ꞵ* = 39.9 (95% *CI:* 33.7–46.1), *P* < 0.001]

Among the 326 studies that provided data for this meta-analysis, it was not possible to identify the regional health directorates in 13 (4.0%) of the studies. The remaining 313 studies were reported from 17 regional health directorates. Most of the studies were carried out in Agnéby-Tiassa Mé, Tonpki, Abidjan 1 Grands ponts, Cavally-Guemon and Poro Tchologo Bagoué, where 96 (30.7%), 53 (16.9%), 36 (11.5%), 24 (7.7%) and 36 (11.5%) studies were reported, respectively. Studies with sample sizes exceeding 1000 individuals were predominantly school-based and carried out between 1994 and 2023 (Fig. 2). The prevalence of schistosomiasis decreased from 1974 to 2023. The shape of funnel plot of the studies was asymmetric, indicated the presence of publication bias, which was statistically evidenced by Egger’s test (*P* < 0.001) (Fig. 3).

### Prevalence of schistosomiasis

There was considerable heterogeneity in the prevalence of schistosomiasis among the included studies (*I*^2^ = 99.6%, Cochran’s *Q P* < 0.001) (Table 1). Hence, a random effect model was employed and revealed that the pooled prevalence of schistosomiasis among all age categories in Côte d’Ivoire was 26.1% (95%* CI:* 24.2–27.9%).

**Table 1:** Subgroup prevalence estimate for schistosomiasis in Côte d’Ivoire

Variables	Subgroups analysis	N articles	N examined	Prevalence (%)	95%* CI*	*I*^2^ (%)	*P*-value
Overall		326	279,340	26.1	24.2–27.9	99.6	< 0.001
Study decades	1974–1983	31	3,164	31.9	19.5–44.3	99.1	< 0.001
	1984–1993	3	1,283	32.7	6.4–59.1	99.2	< 0.001
	1994–2003	30	16,155	66.5	53.2–79.8	99.8	< 0.001
	2004–2013	97	159,479	27.3	24.1–30.5	99.5	< 0.001
	2014–2023	165	99,259	15.0	12.7–17.1	99.3	< 0.001
Regional health directorates	Abidjan 1 Grands ponts	36	5,097	2.9	1.3–4.6	91.1	< 0.001
	Abidjan 2	5	1,305	4.4	− 0.1 to 8.9	72.9	0.011
	Agnéby-Tiassa Mé	96	24,946	34.6	29.3–39.8	99.6	< 0.001
	Bélier	11	5,206	26.4	20.1–32.8	98.2	< 0.001
	Tonkpi	53	33,858	50.1	45.0–55.2	98.9	< 0.001
	Cavally-Guemon	24	2,114	11.6	8.0–15.1	85.7	< 0.001
	DRS	13	190,158	16.8	11.3–22.2	99.9	< 0.001
	Gbêkê	12	1,360	13.1	5.7–20.6	96.1	< 0.001
	Gboklé Nawa San Pedro	3	1,080	2.5	− 0.1 to 5.1	90.3	0.002
	Gôh	2	490	8.5	− 4.0 to 21.0	86.4	0.007
	Hambol	14	1,884	4.6	2.3–7.0	91.0	< 0.001
	Haut-Sassandra	7	1170	13.6	0.3–26.9	98.0	< 0.001
	Indénié Djuablin	4	1,263	10.2	3.2–17.3	97.6	< 0.001
	Kabadougou Bafing Folon	2	200	18.2	− 16.1 to 52.5	97.8	0.009
	Marahoué	7	3,210	11.2	7.5–14.9	92.5	< 0.001
	Poro Tchologo Bagoué	36	5,969	13.2	9.9–16.5	95.7	< 0.001
	Sud Comoé	1	30	13.3	3.8–30.7	0.0	–
Study population	Schoolchildren	226	254,954	28.7	26.4–31.0	99.6	< 0.001
	Community	97	23,866	19.5	17.1–21.8	98.3	< 0.001
	Pre-schoolchildren	3	520	16.6	4.0–29.1	93.7	< 0.001

*CI* confident interval, *DRS* Studies carried out in differents regional health directorates, *N* number

### Subgroup analysis

Table 1 summarises subgroup analyses that revealed stark differences in schistosomiasis prevalence between regional health directorates, time of surveys (decadal periods), study populations and schistosome species. Stratified by the decade of publication, approximately half of the articles (50.6%) were reported in the last decade (2014–2023). The highest prevalence of schistosomiasis was obtained between 1994 and 2003 (66.5%; 95% *CI:* 53.2–79.8%), while the lowest prevalence was observed between 2014 and 2023 (15.0%; 95%* CI:* 12.7–17.1%). Stratified by regional health directorates, the highest prevalence of schistosomiasis was observed in Tonkpi regional health directorate (50.1%; 95% *CI: *45.0–55.2%), located in the western part of Côte d’Ivoire. The lowest prevalence of schistosomiasis was reported in Gboklé Nawa San Pedro (2.5%, 95% *CI:* − 0.1–5.1%) and Abidjan 1 Grands ponts (2.9%, 95% *CI:* 1.3–4.6%). Stratified by study population, the pooled prevalence of schistosomiasis was significantly higher among school-aged children (28.7%, 95%* CI:* 26.4–31.0%) compared to preschool-aged children (16.6%, 95%* CI:* 4.0–29.1%).

The prevalence of *S. haematobium* and *S. mansoni*, stratified by regional health directorate is summarised in Table 2. One third of the surveys pertaining to urogenital schistosomiasis were conducted in Agneby-Tiassa Mé regional health directorate (35.1%, 80/228). The pooled prevalence of *S. haematobium* in Côte d’Ivoire was 19.0% (95% *CI:* 17.3–20.8%) (Table 2). The highest prevalence of S. haematobium was 42.8% (95% *CI:* 36.6–49.2%), reported in Abidjan 1 Grands ponts. The largest number of *S. mansoni* surveys were found in Agnéby-Tiassa Mé (77 articles) and Tonkpi (51 articles) regional health directorates. The pooled prevalence of *S. mansoni* was 20.0% (95% *CI:* 18.1–21.9%) across Côte d’Ivoire (Table 2). The highest S. mansoni prevalence was reported in Tonkpi regional health directorate (45.5%, 95% *CI:* 40.4–50.7%).

**Table 2:** Prevalence of *S. haematobium* and *S. mansoni* stratified by regional health directorates

Regional health directorates	*S. haematobium*				*S. mansoni*			
	N articles	N examined	Prevalence (%)	95% *CI*	N articles	N examined	Prevalence (%)	95% *CI*
Abidjan 1 Grands ponts	1	250	42.8	36.6–49.2	35	4,847	0.7	0.2–1.1
Abidjan 2	1	108	0.0		5	1,305	4.4	− 0.1 to 8.9
Agnéby-Tiassa Mé	80	19,204	28.6	24.2–33.0	77	20,964	22.4	18.4–26.3
Bélier	11	5,206	26.4	20.1–32.8	10	4,767	2.6	1.3–3.9
Tonkpi	31	4,473	18.9	14.3–23.5	51	32,831	45.5	40.4–50.7
Cavally-Guemon	24	2,164	2.6	1.1–4.1	24	2,016	11.5	7.9–15.0
DRS	6	141,844	14.1	7.3–21.0	10	172,437	17.4	11.1–23.8
Gbêkê	12	1,357	12.6	5.4–19.9	9	751	1.1	− 0.1 to 2.3
Gboklé Nawa San Pedro					3	1,080	2.5	− 0.1 to 5.1
Gôh					2	490	8.5	− 4.0 to 21.0
Hambol	14	1,966	2.3	0.8–3.7	10	1319	6.2	2.7–9.6
Haut-Sassandra	6	810	20.2	7.9–32.5	5	556	1.2	− 0.9 to 3.2
Indénié Djuablin					4	1,263	10.2	3.2–17.3
Kabadougou Bafing Folon					2	200	18.2	− 16.1 to 52.5
Marahoué	6	2,850	9.7	6.5–12.9	7	3,210	8.1	5.0–11.1
Poro Tchologo Bagoué	36	6,133	5.4	3.6–7.2	35	5,825	10.3	7.6–12.9
Sud Comoé					1	30	13.3	3.8–30.7
Total	228	186,365	19.0	17.3–20.8	290	253,891	20.0	18.1–21.9

*CI* confident interval, *DRS* Studies carried out in differents regional health directorates, *N* number

**Fig. 4:**
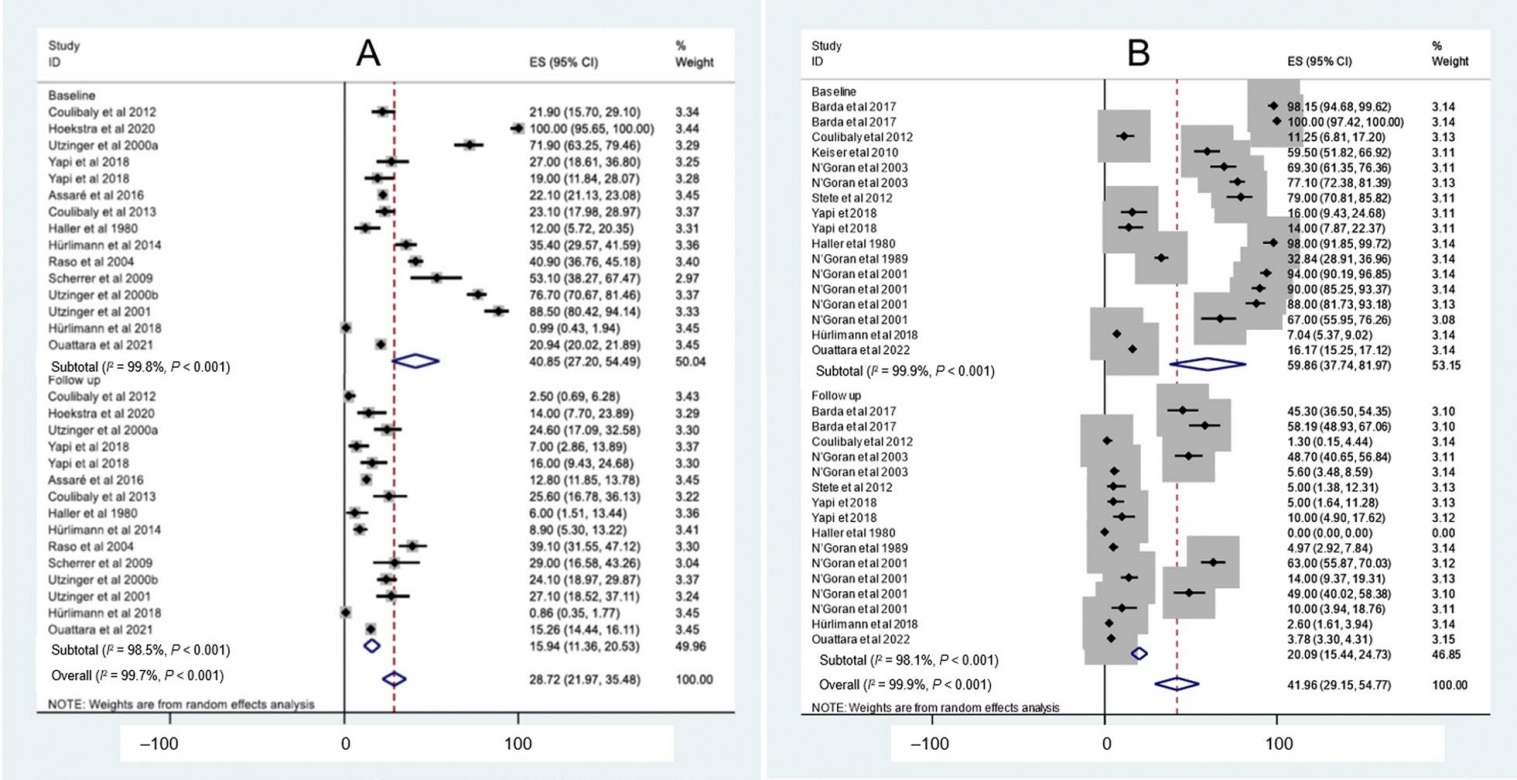
Forest plot of prevalence of *Schistosoma* species at baseline and follow-up of control studies conducted across Côte d’Ivoire.** A** Intervention for *S. mansoni* control. **B** Intervention for *S. haematobium* control

The reported control or specific interventions for *S. mansoni* between 1974 and 2023 are shown in Table 3. There were 19 studies; 11 pertained to mass drug administrations (MDA), five were clinical trials, two focused on improved sanitation and one to MDA combined with WASH. The first intervention was conducted in 1980 in Mopé, while the most recent study pertaining to *S*. *mansoni* was implemented from October 2018 to 2019 in three villages of Taabo (Agnéby-Tiassa Mé regional health directorate). There were three studies without follow-up for *S. mansoni* prevalence, resulting in 16 studies for further analysis. The pooled prevalence of *S. mansoni* decreased from 40.9% (95% *CI:* 27.2–54.5%) to 15.9% (95% *CI:* 11.4–20.5%) (Fig. 4A).

**Table 3:** Reported interventions for schistosomiasis mansoni control from 1974 to 2023 in Côte d’Ivoire

Author	Setting	Study population	Type of intervention	Diagnostic method	Date of baseline	Date of follow-up	Baseline sample size	Baseline *S. mansoni* prevalence	Follow-up sample size	Follow-up *S. mansoni* prevalence
Coulibaly et al. 2012	2 villages in Azaguié	< 6 years	Clinical trial	KK	June 2011	November 2011	160	21.9	160	2.5
Coulibaly et al. 2017	5 villages of Azaguié	2–5 years	Clinical trial	KK	November 2014	February 2015	660	24.0	143	
Coulibaly et al. 2017	5 villages of Azaguié	5–15 years	Clinical trial	KK	November 2014	February 2015	225	80.0	174	
Hoekstra et al. 2020	3 villages of Taabo	5–17 years	Clinical trial	KK	October 2018	Janvier 2019	83	100.0	83	14.0
Utzinger et al. 2000a	Fagnampleu	6–15 years	Clinical trial	KK	December 1998	June 1999	128	71.9	128	24.6
Yapi et al. 2018	Blanfla	Community	Productive sanitation	KK	June 2014	July 2016	100	27.0	100	7.0
Yapi et al. 2018	North Diacohou	Community	Productive sanitation	KK	June 2014	July 2016	100	19.0	100	16.0
Adoubryn et al. 2012	Biankouma	4–15 years	MDA	KK	2006	2007	386	35.5		
Agbaya et al. 2004	Agboville	4–15 years	MDA	KK	February 2001	June 2001	360	10.0		12.5
Assaré et al. 2016	4 regions of westerrn Côte d’Ivoire	9–12 years	MDA	KK	December 2011 to January 2012	May 2013	7011	22.1	4667	12.8
Coulibaly et al. 2013	2 village in Azaguié	< 6 years	MDA	KK	August 2011	November 2011	242	23.1	86	25.6
Haller et al. 1980	Mopé	5–15 years	MDA	KK	1980	1980	86	12.0	73	6.0
Hürlimann et al. 2014	Niabé (Abengourou)	4–15 years	MDA	KK	December 2012	May 2013	257	35.4	219	8.9
Raso et al. 2004	Zouatta II	Community	MDA	KK	May 2002	June 2002	545	40.9	161	39.1
Scherrer et al. 2009	Douimbly and Zouatta II	4–13 years	MDA	KK	June 2007	July 2007	49	53.1	49	29.0
Utzinger et al. 2000b	Fagnampleu	6–14 years	MDA	KK	November 1998	December 1998	253	76.7	253	24.1
Utzinger et al. 2001	Gbatongouin and Mélapleu	7–14 years	MDA	KK	November 1997	February 1998	96	88.5	96	27.1
Hürlimann et al. 2018	5 villages South Center of Côte d’Ivoire	Community	MDA and WASH	KK	July 2011	August 12	810	1.0	810	0.9
Ouattara et al. 2021	75 Locations Western Côte d’Ivoire	9–12 years	MDA	KK	December 2011 to January 2012	2016	7410	20.9	7223	15.3

*KK* Kato-Katz technique, *MDA* mass drug administration, *WASH* water, sanitation, and hygiene

The reported intervention for the control of *S. haematobium* between 1974 and 2023 are summarised in Table 4. There were 19 studies, including nine clinical trials, seven MDA, two improved sanitation and one MDA plus WASH. The first study focusing on *S. haematobium* control was carried out in 1980 in Mopé (Agnéby-Tiassa Mé regional health directorate), while the most recent studies took place from November 2015 to 2019 in 64 villages in the northern and central parts of Côte d’Ivoire. There were three studies without follow-up of *S. haematobium* prevalence. Hence, 16 intervention studies were included in the analysis. The pooled prevalence of *S. haematobium* decreased from 59.9% (95% *CI:* 37.7–82.0%) to 20.1% (95% *CI:* 15.4–24.7%) (Fig. 4B).

**Table 4:** Reported interventions for schistosomiasis haematobium control from 1974 to 2023 in Côte d’Ivoire

Author	Setting	Study population	Type of intervention	Diagnostic method	Date of baseline	Date of follow-up	Baseline sample size	Baseline *S. haematobium *prevalence	Follow-up sample size	Follow-up *S. haematobium* prevalence
Barda et al. 2017	4 villages of Adzopé	2–5 years	Clinical trial	Urine filtration	November 2015	May 2016	162	98.2	128	45.3
Barda et al. 2017	4 villages of Adzopé	6–15 years	Clinical trial	Urine filtration	November 2015	May 2016	141	100.0	122	58.2
Coulibaly et al. 2018	5 villages of Adzopé	2–5 years	Clinical trial	Urine filtration	November 2015	February 2016	628	29.6	157	
Coulibaly et al. 2018	5 villages of Adzopé	6–15 years	Clinical trial	Urine filtration	November 2015	February 2016	356	45.7	166	
Coulibaly et al. 2012	2 village in Azaguié	< 6 years	Clinical trial	Urine filtration	June 2011	November 2011	160	11.3	160	1.3
Keiser et al. 2010	Guéssiguié	Aug 16	Clinical trial	Urine filtration	November 2008	December 2008	173	59.5	83	
N’Goran et al. 2003	Taabo Village	5–15 years	Clinical trial	Urine filtration	November 2000	July 2001	156	69.3	156	48.7
N’Goran et al. 2003	Taabo Village	5–15 years	Clinical trial	Urine filtration	November 2000	June 2001	354	77.1	354	5.6
Stete et al. 2012	Grand Moutcho	7–15 years	Clinical trial	Urine filtration	March 2010	June 2010	124	79.0	80	5.0
Yapi et al. 2018	Blanfla	Community	Productive sanitation	Urine filtration	June 2014	July 2016	100	16.0	100	5.0
Yapi et al. 2018	North Diacohou	Community	Productive sanitation	Urine filtration	June 2014	July 2016	100	14.0	100	10.0
Haller et al. 1980	Mopé	5–15 years	MDA	Urine filtration	1980	1980	86	98.0	73	0.0
N’Goran et al. 1989	N’Guessan Pokoukro	Community	MDA	Urine filtration	1989	1989	545	32.8	342	5.0
N’Goran et al. 2001	Taabo Village	4–15 years	MDA	Urine filtration	May 1997	Octber 1997	222	94.0	190	63.0
N’Goran et al. 2001	Bodo	4–15 years	MDA	Urine filtration	May 1997	Octber 1997	236	90.0	203	14.0
N’Goran et al. 2001	Batera	4–15 years	MDA	Urine filtration	May 1997	Octber 1997	137	88.0	122	49.0
N’Goran et al. 2001	Assinzé	4–15 years	MDA	Urine filtration	May 1997	Octber 1997	90	67.0	73	10.0
Hürlimann et al. 2018	5 villages South Center of Côte d’Ivoire	Community	MDA and WASH	Urine filtration	July 2011	August 2012	810	7.0	810	2.6
Ouattara et al. 2022	64 villages of Northern and Central Côte d’Ivoire	Community	MDA	Urine filtration	November 2015	2016–2019	6092	16.2	5689	3.8

*MDA* mass drug administration, *WASH* water, sanitation, and hygiene

### Risk factors for schistosomiasis

**Fig. 5:**
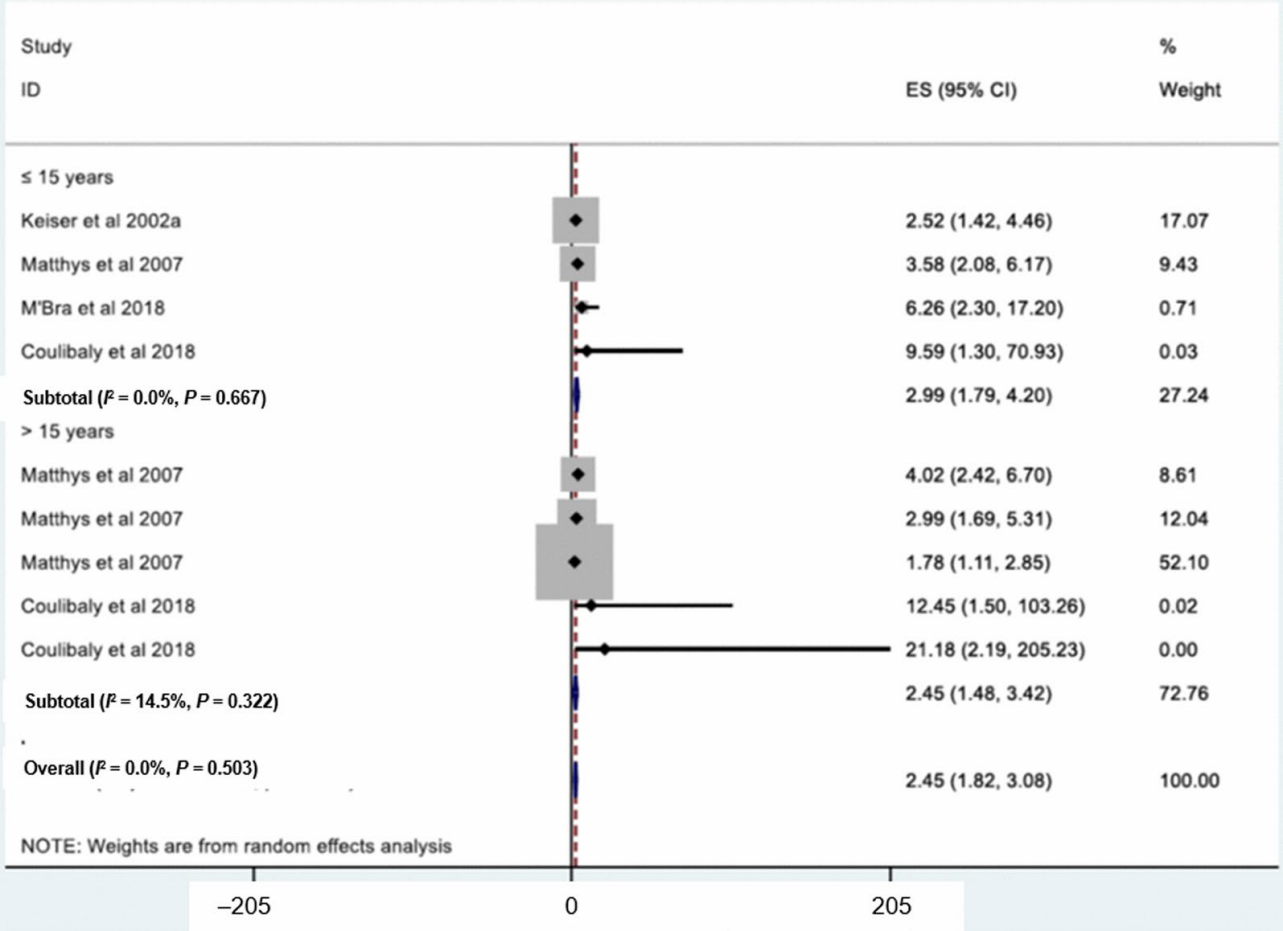
Forest plot for odds ratio of the association of age group with *S. mansoni*. Age group-reference: 6–10 years

**Fig. 6:**
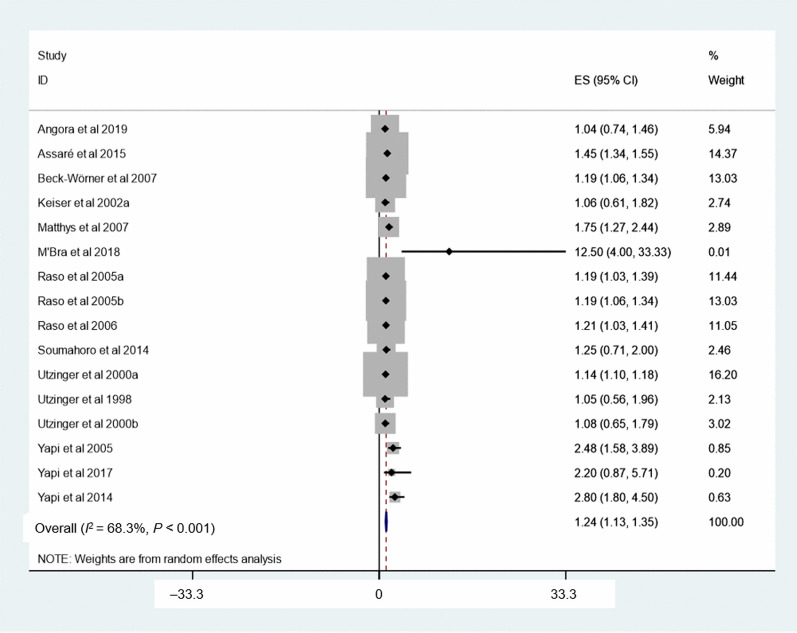
Forest plot for odds ratio of the association sex with schistosomiasis. Sex reference: Female

The relationship between age group and *S. mansoni* is shown in Fig. 5. Four studies were included to determine associations between age and *S. mansoni*. Children aged above 15 years had a higher odds of *S. mansoni* infection than their younger counterparts aged 6–10 years (*OR* = 2.45, 95% *CI:* 1.48–3.42). For the assessment of the relationship between sex and schistosomiasis, 15 studies were included. High heterogeneity was observed (*I*^2^ = 57.3%, *P* < 0.001), and hence, a meta-analysis with a random effect model was performed. There was a statistically significant association between sex and schistosomiasis with males at higher odds of *Schistosoma* infection than females (*OR* = 1.24, 95% *CI:* 1.13–1.35) (Fig. 6).

**Fig. 7:**
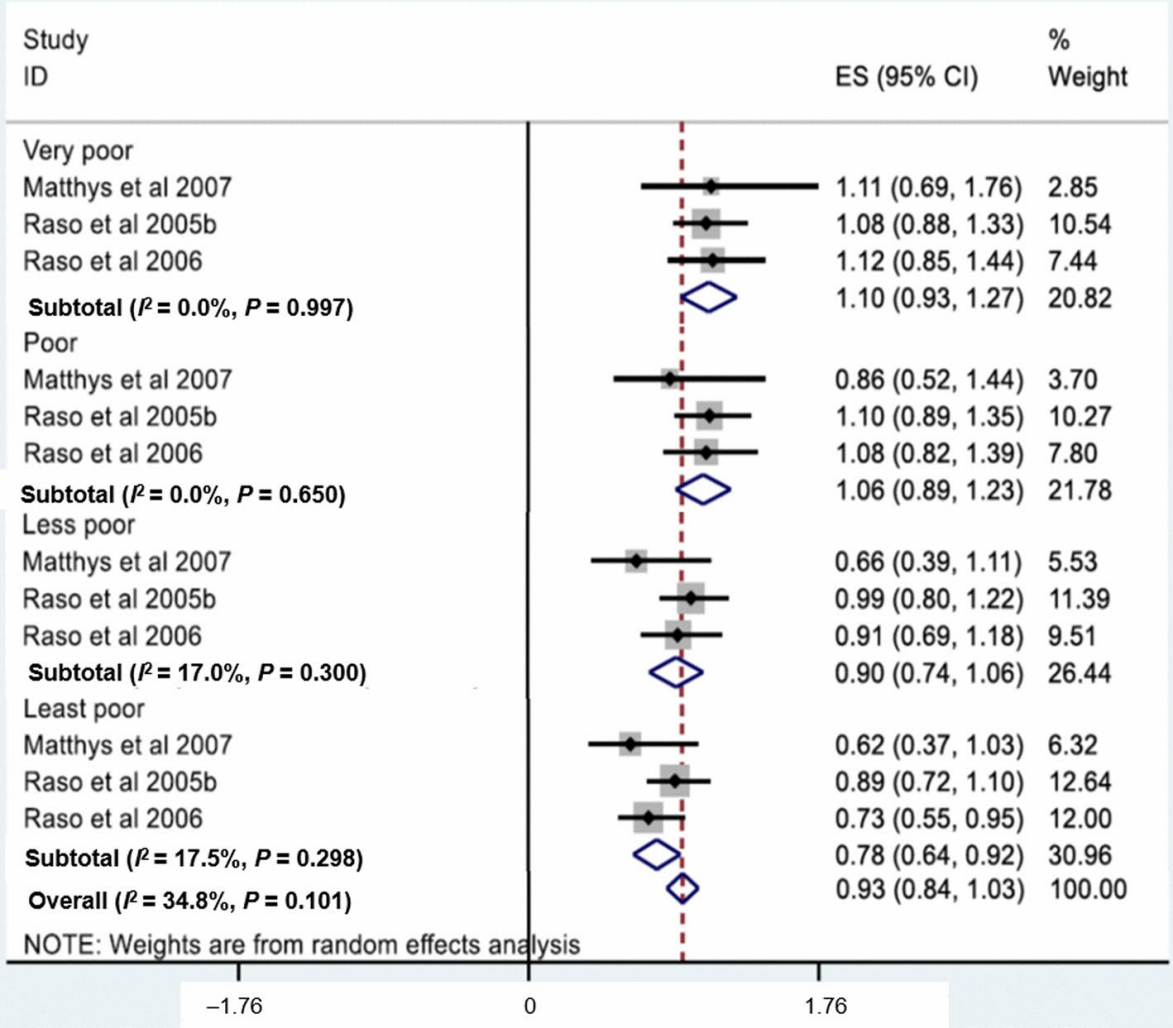
Forest plot for socio-economic status with schistosomiasis

Three studies were included to determine the association between socioeconomic status and schistosomiasis. Least poor individuals were less likely to be infected with *Schistosoma* spp. compared to the poorest quintile (*OR* = 0.78, 95% *CI:* 0.64–0.92) (Fig. 7). The odds of infection among poor individuals grouped into less poor, poor and very poor quintiles were 0.90 (95% *CI:* 0.74–1.06), 1.06 (95% *CI: *0.89–1.23) and 1.10 (95%* CI:* 0.93–1.27), respectively, when compared to most poor as reference. The pooled *OR* showed that there was no significant association (*OR* = 0.93, 95% *CI:* 0.84–1.03).

**Fig. 8:**
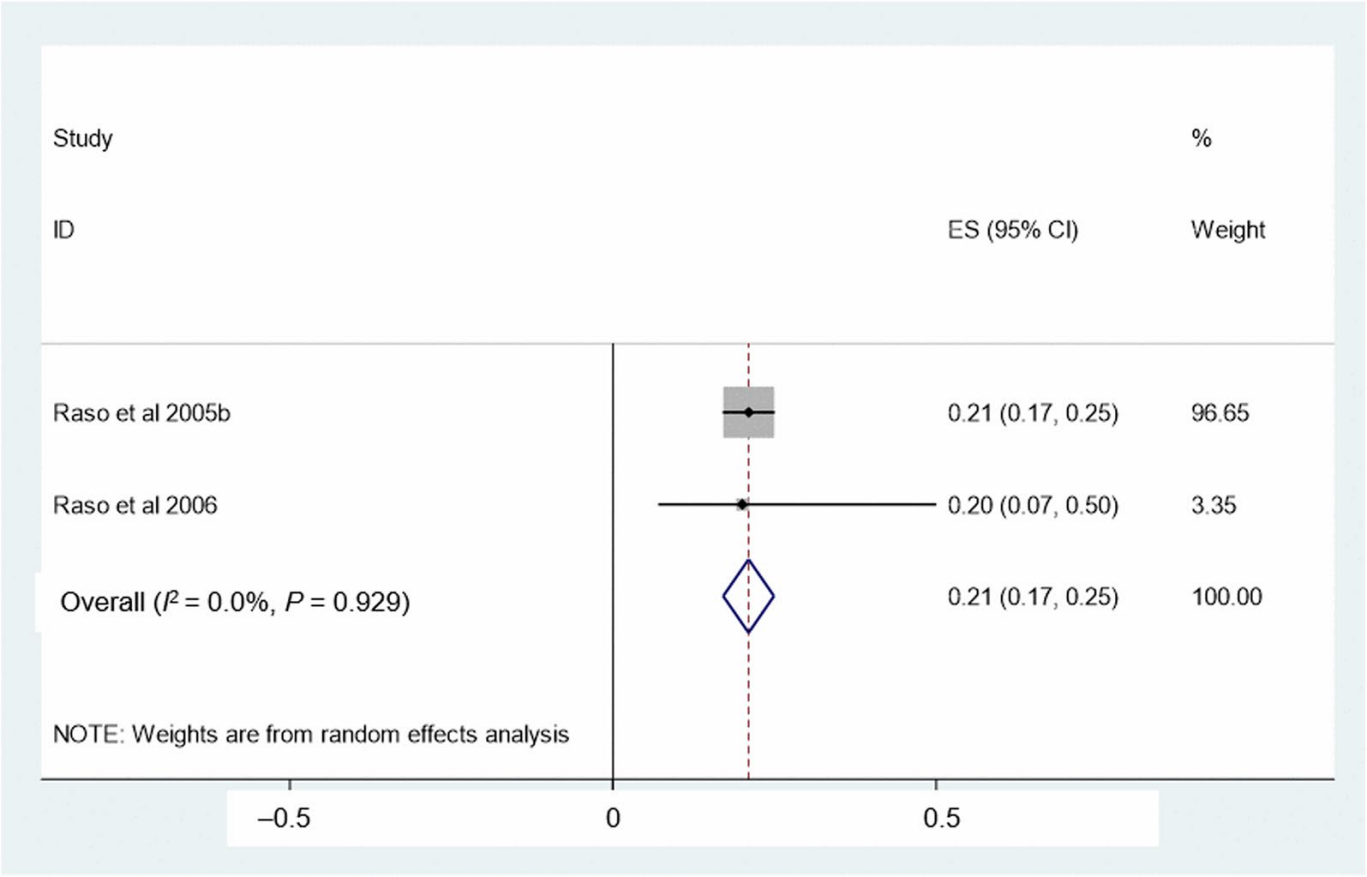
Relationship between altitude and schistosomiasis. Reference altitude: < 400 m

The relationship between altitude and schistosomiasis is shown in Fig. 8. The pooled *OR* showed that the odds of being infected with schistosome was much lower among people living at higher altitude (> 400 m) compared to those living at lower altitude (*OR* = 0.21, 95%* CI:* 0.17–0.25).

## Discussion

We present the first systematic review and meta-analysis regarding the prevalence of human schistosomiasis in Côte d’Ivoire, including spatial and temporal trends and underlying risk factors. Most of the data stem from school-aged children and we found a pooled prevalence of schistosomiasis in Côte d’Ivoire of 26.1%. A recent systematic review and meta-analysis of schistosomiasis in school-aged children in Ethiopia found a slightly higher pooled prevalence of 28.8% [36]. In contrast, a systematic review coupled with a geospatial analysis assessing the spatial distribution of schistosomiasis and treatment needs in sub-Saharan Africa, reported significantly lower prevalence of schistosomiasis in Côte d’Ivoire (17.4%) [37]. In 2011, a geospatial analysis estimating the prevalence of schistosomiasis among children and adolescents below 20 years in West Africa reported a prevalence of 19.5% for Côte d’Ivoire [38]. A considerably lower prevalence of schistosomiasis was observed in national parasitological surveys conducted in Côte d’Ivoire (8.9%) and Kenya (2.1%) [39, 40]. The pooled prevalence of schistosomiasis in Côte d’Ivoire was significantly lower than results of a systematic review done in Zambia (47.5%) and a national survey carried out in Sierra Leone (42.2%) [41, 42]. The difference in the reported countrywide schistosomiasis prevalence estimates might be due to differences in study methodologies, WASH indicators, socioeconomic status, environmental characteristics of the countries and MDA coverage [7, 12, 43, 44].

Our findings showed that the pooled prevalence of schistosomiasis in Côte d’Ivoire was approximately halved from 1974 to 2023. In addition, the analysis of the reported intervention studies for the control of *S. haematobium* and *S. mansoni* revealed that urogenital and intestinal schistosomiasis were two-fold lower in recent years compared to the situation towards the end of the last millennium. The most likely explanation of this observation is that the national schistosomiasis control programme (PNLMTN-CP), emphasising MDA with praziquantel targeting school-aged children, showed success. In addition, water bodies in some locations have been treated with the molluscicide niclosamide, while communities in schistosomiasis endemic settings were sensitized to follow WASH strategies in order to prevent the disease [45, 46].

Our results revealed highest prevalence rates of schistosomiasis in the Tonkpi regional health directorate (50.1%). This observation confirms results of previous studies carried out in Côte d’Ivoire, which reported *Schistosoma* infection prevalence ranging from 50 to 100% [47–49]. Historically, intestinal schistosomiasis due to *S. mansoni* was the predominant schistosome species in this region, explained by environmental factors providing suitable conditions for the development of *Biomphalaria pfeifferi*, the intermediate host snail of *S. mansoni*. Moreover, most people in western Côte d’Ivoire practiced open defecation and several water contact activities in freshwater bodies such as bathing, washing clothes, crossing rivers, fishing and fetching water for domestic and agricultural use, which drive environment contamination and expose people to schistosome infection [19, 50].

Our subgroup analyses showed that schistosomiasis prevalence in preschool-aged children was higher than 10% (i.e. 16.6%). Moderate and high prevalence of schistosomiasis in preschool-aged children have been reported in previous epidemiological assessment in Côte d’Ivoire (31.4%), Ethiopia (14.6%) and Tanzania (60.6%) [51–53]. A systematic review and meta-analysis quantifying schistosomiasis infection in preschool-aged children in sub-Saharan Africa for the past 20 years revealed that the pooled prevalence of the disease was 19% [54]. These findings provide an evidence-base to include preschool-aged children in a comprehensive package of schistosomiasis control. Our findings also showed that the prevalence of schistosomiasis is higher among school-aged children compared to preschool-aged children. This observation corroborates with other studies from sub-Saharan Africa. Indeed, the prevalence of *Schistosoma* infection increases from early childhood with a peak usually observed at the age of 8–15 years, depending on the overall intensity of infection and decreases in adolescents and adulthood depending on setting-specific socioeconomic and behavioural factors [24, 52, 55, 56].

Findings on the analysis on relationship between age and schistosomiasis showed that there was strong association between age and schistosomiasis infection with older children at higher odds of infection compared to their younger counterparts. This observation might be explained by the characteristic of the study population. Indeed, in the study areas, older children practiced fishing and swimming in freshwater bodies and were involved in farming with their parents in irrigation schemes [57–60]. Hence, they were more exposed to schistosome cercariae than their younger counterparts.

The results of our systematic review and meta-analysis showed that males were more likely infected with schistosomes than females (*OR* = 1.24). A systematic review investigating the association between schistosomiasis pooled prevalence among Ethiopian school-aged children found that boys were more likely to be infected with schistosomes than girls (*OR *= 1.6) [36]. In addition, in a recent systematic review analysing 123 studies with prevalence and intensity of *S. haematobium* and *S. mansoni* infection showed that males were significantly more likely to be infected than females [61]. One plausible explanation is that males had higher frequency of contact with contaminated freshwater bodies and engaged in outdoor activities (e.g. fishing, swimming and irrigation farming) more often than females [14, 62, 63]. Other explanations could be religious and sociocultural reasons that limit water contact activities of females [64], and the adventurous nature of males who spent more time away from their homes than females [65].

The findings of our study further revealed that least poor individuals were less likely to have an infection with schistosome compared to the poorest quintile (*OR* = 0.78). A previous study conducted in Uganda evaluating the association between socioeconomic status and schistosomiasis reported that the odds of being infected for the lowest wealth quintile was 10.4 times higher compared to the highest wealth quintile (*OR* = 10.42). [66]. These data indicate that wealthy persons were less likely to have *Schistosoma* infection. A plausible explanation for this finding is that wealthy persons have limited contact with infected water bodies and have better access to health services and praziquantel treatment. This observation confirms that schistosomiasis is a poverty-related disease and improving the socioeconomic status of communities could help to decrease significantly the burden of schistosomiasis.

Our study showed that the odds of being infected with schistosome were much lower among people living at higher altitude (> 400 m) compared to those living at lower altitudes (*OR* = 0.21). A negative association between altitude and schistosomiasis prevalence was also found in the western part of Côte d’Ivoire, the western part of Ethiopia, the south-western part of Tanzania and the north-eastern part of the Democratic Republic of the Congo [28, 29, 67–69]. It is suggested that higher altitude leads to higher water velocity and cooler temperature of water bodies that present unsuitable habitat conditions for schistosomiasis intermediate host snails.

## Strengths and limitations

Our study has several limitations that are offered for consideration. First, the sample size in some studies was low, which in turn could affect the estimated pooled prevalence of schistosomiasis and the validity of the obtained results. Second, no prior systematic review and meta-analysis assessing schistosomiasis prevalence and risk factors in Côte d’Ivoire was available for comparison. Third, the inclusion of studies using more sensitive diagnostic methods such as point-of-care circulating cathodic antigen urine cassette test for *S. mansoni* could result in higher pooled prevalence estimates of schistosomiasis. Fourth, data from 9 studies overlapped in different regional health directorates, which limit the subgroup analysis at regional health directorate scale. Lastly, data available from PNLMTN–CP and other non-governmental agencies were not considered because we only used data from published articles for replicability reason. Hence, care is indicated while interpreting the results of our meta-analysis.

Strengths of our study are that we adhered to the PRISMA guidelines, employed random effect models for meta-analysis and covered a large time frame of half a century. The marked decline of the prevalence of human schistosomiasis over the past 50 years in Côte d’Ivoire is encouraging. Data presented here will be shared, uploaded and curated by the Global Neglected Tropical Diseases database [70] for continued update and refinement of schistosomiasis geostatistical analysis at different spatial scales across Africa.

## Conclusion

The systematic review and meta-analysis presented here showed moderate prevalence of human schistosomiasis in Côte d’Ivoire. Moderate prevalence was also reported among preschoolers. Hence, the study highlights the need to include preschool-aged children in control programmes and the availability of a paediatric formulation of praziquantel is welcomed. The main risk factors for schistosomiasis are age (> 15 years), male gender, living in low-income households and low altitude (< 400 m). The quality of the sampling method is an important parameter that should be considered in epidemiological investigations of schistosomiasis. We encourage public health decision makers in Côte d’Ivoire to improve surveillance and tailor control efforts to high-risk communities, in order to further reduce the burden of the disease. Future studies should assess the prevalence, intensity and burden of schistosomiasis, the intermediate host snails and species abundance and distribution in the context of climate change and continuous efforts with integrated control approaches.

## Supplementary Information

Supplementary Material 1.

Supplementary Material.2.

Supplementary Material 3.

## Data Availability

The data used in current manuscript are available as supplementary material.

## Abbreviations

*CI*: Confidence interval
CNRA: Centre National de Recherche Agronomique de Côte d’Ivoire
DRS: Studies carried out in different regional health directorates
HDSS: Health and demographic surveillance system
JBI: Joanna briggs institute
KK: Kato-Katz technique
MDA: Mass drug administration
*n*: Number
*OR*: Odds ratio
*P*: *P*-value
PICOS: Participants, interventions, comparisons, outcomes, and study design
PNLMTN–CP: Programme national de lutte contre les maladies tropicales négligées à chimiothérapie préventive
PRISMA: Preferred reporting items for systematic reviews and meta-analysis
WASH: Water, sanitation and hygiene
WHO: World Health Organization
